# Effect of triple-frequency sono-germination and soaking treatments on techno-functional characteristics of barley

**DOI:** 10.1016/j.ultsonch.2025.107231

**Published:** 2025-01-12

**Authors:** Tabussam Tufail, Huma Bader Ul Ain, Jawad Ashraf, Muhammad Safiullah Virk, Zahoor Ahmed, Mokhtar Dabbour, Tawfiq Alsulami, Suleiman Althawab, Bin Xu

**Affiliations:** a School of Food and Biological Engineering Jiangsu University, Zhenjiang, Jiangsu 212013, China; b University Institute of Diet and Nutritional Sciences, The University of Lahore, Pakistan; c School of Food Science and Engineering, Yangzhou University, Yangzhou, China; d Human Nutrition and Dietetics, School of Food and Agricultural Sciences, University of Management and Technology, Lahore, Pakistan; e Department of Agricultural and Biosystems Engineering, Faculty of Agriculture, Benha University, P.O. Box 13736, Moshtohor, Qaluobia, Egypt; f Department of Food Science & Nutrition, College of Food and Agricultural Sciences, King Saud University, Riyadh 11451, Saudi Arabia

**Keywords:** Barley varieties, Triple-frequency ultrasound, Germination, Soaking, GABA synthesis, Techno-functional properties, Computational analysis

## Abstract

This research aimed to evaluate the effect of triple-frequency ultrasound treatment (TFUT), germination (GE), and traditional soaking (TS) methods on the nutritional and techno-functional properties of two different barley varieties, including ZQ2000 and XMLY22. Both ZQ2000 and XMLY22 varieties exhibited the highest total phenolic content (TPC) with 840.73 ± 23.59 μg of GAE/g DW and 720.33 ± 30.56 μg of GAE/g DW, and total flavonoid content (TFC) with 520.79 ± 23.45 μg of QUE/g DW and 420.84 ± 19.80 μg of QUE/g DW, respectively. Enzyme activities, such as peroxidase (POD) and polyphenol oxidase (PPO), were notably elevated, indicating enhanced defense mechanisms. The study also found increased γ-Aminobutyric Acid (GABA) levels and antidiabetic potential through inhibition of α-amylase and α-glucosidase enzymes. Further, gene expression analysis revealed differential regulation of phenylpropanoid pathway genes, contributing to the bioactive compound enhancement. Strong intermolecular interactions were observed in both ZQ2000 and XMLY22 samples subjected to TFUT, GE, TFUT + GE, and TS, as validated by FTIR and molecular docking analyses. The structural configuration of two barley types, ZQ2000 and XMLY22, was determined using Fourier transform infrared (FTIR) spectroscopy, which indicated an increase in *α*-helix and *β*-sheet conformation and a decrease in random coil conformation in samples treated with TFUT + GE. Moreover, SEM observation provides convincing evidence that TFUT + GE improves and speeds up the breakdown of ZQ2000′s internal structures. Conclusively, this study suggests that the combination of ultrasound and germination treatments significantly enhances the functional properties of barley, making it a promising method for creating health-enhancing barley-based products offering potential applications in functional food development.

## 1 Introduction

Barley (*Hordeum vulgare* L.) is one of the oldest cultivated grains, valued for its adaptability to diverse climates and nutritional benefits. It is widely used in food, beverages, and animal feed, offering a rich source of dietary fiber, vitamins, and bioactive compounds [1]. Barley with naked caryopsis is primarily found on the Tibetan Plateau at altitudes of 1400–4700 m, with over 70 % of Tibet’s agricultural areas dedicated to this staple food [2]. Highland barley’s nutritional value, such as fiber, protein, β-glucan, and phenolic compounds, has been studied for its potential advantages in managing hyperlipidemia, diabetes, blood pressure, and atherosclerosis [3]. Furthermore, barley contains gamma-aminobutyric acid (GABA), a nonprotein amino acid that plays a crucial role as an inhibitory neurotransmitter in the central nervous system. It exhibits numerous physiological effects, including hypertension, hypoglycemia, anti-cancer, diuretic, and sedative effects [4], [5]. Depending on species, stage of development, growing environment, and processing conditions, plants naturally contain GABA in modest levels to micromoles per gram normally < 10 to 50 mg/100 g dry weight basis [6], [7]. Depending on species, stage of development, growing environment, and processing conditions, plants naturally contain GABA in modest levels, up to micromoles/gram [6], [7]. Stress application in barley processing involves biochemical including enhancement of nutritional profile and physical changes in the grain [8], [9], [10].

Ultrasound treatment generates cavitation, releasing antioxidants and phenolic compounds and improving digestibility by altering starch and protein structure [7], [11]. Germination, sprouting barley seeds under controlled conditions, activates enzymes, breaking down complex carbohydrates, proteins, and anti-nutrients. This increases nutrient content, improves digestibility, and increases mineral bioavailability [12]. Soaking softens grains, reduces anti-nutrient levels, and improves the texture and cooking properties. The ideal temperature for soaking seeds in water is between 20–25 °C. Several factors, including MDA, CAT, SOD, H2O2, POD, and PPO influence the antioxidant defense and oxidative stress response of barley. These stress applications significantly modify barley’s nutritional and functional properties, making it a more beneficial food source [13]. Barley’s nutritional and functional properties depend on its germination rate and ultrasonic and soaking treatments [14]. Barley seed GABA, Glu, GAD, and GABA-T levels can also be impacted by the treatments mentioned above [7]. Cavitation in barley seeds can be caused by breaking cell membranes and absorbing water, while low-power (20 HZ) ultrasound can speed up hydration but negatively impact malt quality [15], [16]. In contrast to conventional single-frequency ultrasonic devices, the enhanced mechanical interference and cavitation nuclei in triple-frequency ultrasonic treatment can significantly enhance cavitation yields. Currently, the combined impact of TFUT and germination on the nutritional and techno-functional properties of barley varieties (ZQ2000 and XMLY22) remains unexplored.

The primary distinctions in germination rates between ultrasonic and non-ultrasonic treatments include the rate of germination, the mechanical and thermal effects, species-specific effects, and physiological and biochemical reactions [9], [17]. Traditional Soaking seeds before planting can enhance germination by allowing water to enter the seed coat, extracting inhibitors, and promoting it by a few days to a week [18]. The microstructure of barley kernels significantly influences their quality and processing, with firm and soft kernels having different microstructures that affect pearling and malting [19]. Through the application of stress, it has been documented that germination and soaking stimulate enzymes that degrade complex compounds (such as starches and proteins) into simpler, more easily absorbed forms, enhancing the nutritional composition [20]. Germination and soaking of plants reduce anti-nutrients, such as phytic acid, which improves the absorption of minerals and thus increases nutrient production [21].

The application of stress leads to physical and chemical changes, including the softening of grains and the breakdown of starch, which enhance the digestibility of vital nutrients, making them more accessible to the body. Activating plant defense systems through stress applications may enhance antioxidant levels, consequently fostering the production of specific beneficial bioactives, including phenolics [22]. This study explores how TFUT and germination treatments affect the accumulation of bioactive compounds, antioxidant activities, enzymatic responses, and molecular interactions in the ZQ2000 and XMLY22 barley varieties. The main objective of this research was to characterize the different varieties of Barley and especially focus was given to analyze and observe the effect of different treatments including ultrasonication, germination and soaking on the nutritional and functional characteristics of barley, contributing to global efforts in biofortification and processing technologies.

## 2 Material and methods

### 2.1 Procurement of raw material

The barley was procured from the Yangnongpi 9: Jiangsu Ruimu Biotechnology Co., Zhenjiang, China. The following materials and reagents were collected for the experiment. Two barley varieties, ZQ2000 and XMLY22, were selected, screened from the foreign particles, and washed for further use. The reagents, sodium hypochlorite (CAS No. 7681–52-9), hydrochloric acid (CAS No. 7647–01-0), methyl pyridine (1628–89-3), and gamma-aminobutyric acid (GABA) (CAS No. 56–12-2 HPLC grade), were procured from the certified company.

### 2.2 Sample preparation

The first step involved selecting barley grains without impurities or mold, sterilizing them with 1 % hydrogen peroxide/sodium hypochlorite for 30 min, and soaking them in 3 volumes per weight of deionized water at 30°C for 2 h. After that, barley seeds were separated into five groups including untreated barley (UB), triple-frequency ultrasound treatment (TUFT), germination (GE), the combined effect of TFUT + GE and traditional soaking (TS). The first group was simple washed and dried grains with no treatments (UB), the second group was washed, sterilized, and subjected to the triple-frequency ultrasound treatment (TFUT) with the following frequency (20/40/60 KHz) and power (220 W), followed by a 5-sec on/off interval for 30 min. The third group was germinated in a germinator or rotary evaporator at 65 °C for 6 h. The fourth group was treated with combined effect of triple frequency ultrasonication and germination with same above mention conditions named as (TFUT + GE). The fifth group was soacked in water at 25 °C for 12 h. Furthermore, non-germinated seeds were used as controls, whereas germinated seeds were compared to ultrasonicated and germinated seeds. All the samples treated with ultrasonicated, germinated and socked were stored at −80 °C for 12 h. Afterward, samples were freeze-dried for 48 h and ground to a fine powder using an electric crush, and the powder was sifted through a 120-mesh sieve. Samples were coded, packed in zipper bags, and stored at −24 °C for further analysis.

### 2.3 Extraction of phenolics

#### 2.3.1 Determination of total phenolic contents (TPC)

The TPC of barley treated with different conditions was determined according to the method [23], with slight modifications. The study involved reacting barley or gallic acid with Folin-Ciocalteu reagent and Na_2_CO_3_, measuring absorbance intensity at 765 nm, and expressing TPC values as mg GAE/g dry weight basis. Briefly, Gallic acid standard or samples (20 µl) were mixed with 10- fold diluted Folin–Ciocalteu reagent (100 µl) and reacted at room temperature for 1 min followed by incubation with sodium carbonate (80 µl, 75 g/L) for 30 min. The absorbance was recorded at 765 nm with a Tecan Spark 10-M multimode microplate reader (Tecan Group Ltd., Männedorf, Switzerland).

#### 2.3.2 Determination of total flavonoid contents (TFC)

The TFC was determined using the method; briefly, a 96-well plate was filled with 100 µL of distilled water, 10 µL of NaNO_2_ (50 g/L), and 25 µL of the sample or standard solution. 15 μL of AlCl_3_ (100 g/L) was added to the mixture and stored for 5 min. NaOH (1 M) and distilled water were added in identical quantities (50 μL) after 6 min. The Spectra Max i3 was employed to measure absorbance at 510 nm. The results were expressed as mg of QE/g of DW, with quercetin as the standard [24].

### 2.4 Identification and quantification of free phenolic compounds by HPLC-DAD

Free phenolic compounds were analyzed using an Agilent 1260 HPLC-DAD system (Santa Clara, CA, USA) with a ZARBAX SB-C18 column (Agilent, 4.6 × 250 mm, 5 μm). The mobile phase consisted of a 0.1 % (v/v) formic acid solution (solvent A) and an acetonitrile 100 % solution (solvent B). The linear gradient protocol was established as follows: solvent B is present in the following concentrations: 5 %–10 % solvent B for 0–10 min, 10 %–20 % solvent B for 10–15 min, 20 %–38 % solvent B for 15–25 min, 38 %–40 % solvent B for 25–30 min, 40 %–100 % solvent B for 30–31 min, 100 % solvent B for 31–35 min, 100 %–5% solvent B for 35–36 min, and 5 % solvent B for 36–50 min. The column was maintained at a temperature of 30 °C, and the flow rate was 0.8 mL min^−1^. The chromatograms were recorded at 280, 254, 325, and 520 nm [25].

### 2.5 Determination of antioxidant activity

#### 2.5.1 Determination of DPPH activity

The antioxidant activity was performed by the DPPH radical scavenging protocol. A 100 μL sample was combined with 3.9 ml of DPPH solution (6 × 10^−5^ M) and incubated in a dark location at room temperature for 1 h. The absorbance was determined at 517 nm using a SpectraMax i3 spectrophotometer (Molecular Devices, Sunnyvale, CA, USA) after 1 h. The readings were compared to the controls, which contained double-distilled water instead of extract. The extract’s DPPH radical scavenging activity was determined using the Trolox calibration curve and expressed as μg Trolox equivalents (TE)/g DW [24].

#### 2.5.2 Determination of ABTS activity

The method reported by Mehmood et al. (2019) was employed to determine ABTS activity. The ABTS radical stock solution was generated by combining ABTS (7 mM) with K_2_S_2_O_8_ and storing the mixture in the dark for 16 h. The ABTS radical solution was diluted with PBS (pH 7.4) to absorb 0.70 ± 0.02 at 734 nm. After 6 min, the absorbance was measured at 734 nm after an 80 μL barley was reacted with 3.92 ml of ABTS radical working solution in the dark. Trolox and double-distilled water were employed as controls. The ABTS values were quantified as μg of TE/g DW [26].

#### 2.5.3 Determination of FRAP activity

The reducing power of barley powder was assessed using the ferrous-reducing antioxidant power (FRAP) test as mentioned by Sohail et al., (2018). For this purpose, 1 mL barley extract was mixed with 1 mL buffer (pH 6.6) (1 % potassium ferricyanide and 200 mM sodium phosphate), and the mixture was incubated at 50 °C for 20 min. 1 mL of 10 % trichloroacetic acid (TCA) was also added to the mixture and centrifuged by centrifuge machine (Hettich/Germany/Universal 320 R, SN: 0008017–10) at 3000 rpm for 5 min. Additionally, 1 mL of supernatant was combined with 1 mL of distilled water and 0.1 mL of ferric chloride (0.1 %). The combination was utilized to observe the absorbance at 700 nm. For calibration, FeSO_4_·7H_2_O (100–1,000 µM) aqueous solutions were used, with values given as micromoles of Fe (II) per gram.

### 2.6 Determination of enzyme activity

#### 2.6.1 Determination of SOD, POD, and PPO activities

The xanthine oxidase (EC 1.1.3.22) test, the predominant technique, was conducted to analyze SOD activity according to Sigma-Aldrich guidelines (Bergmeyer 2012). The resulting absorbance was measured for 1 min at 10-sec intervals using a Shimadzu UV-160A UV–VIS spectrophotometer (Shimadzu, Japan) at 550 nm using 1 cm glass cuvettes. Barley samples were homogenized in phosphate buffer to extract enzymes for PPO and POD measurements. After introducing a substrate like catechol, absorbance at 420 nm was measured to determine activity. The rate of quinone formation correlates with PPO activity, and POD activity was measured by the oxidation of substrates such as guaiacol, with absorbance changes monitored at 470 nm. In the end, microplate spectrophotometric techniques were employed to measure enzyme activity, offering advantages such as reduced sample volume and simultaneous analysis of multiple samples [27].

#### 2.6.2 Determination of MDA, CAT, and H_2_O_2_ activities

All the barley seeds (ultrasonicated, germinated and soacked) were pre-treated for sample preparation by soaking in distilled water at 0 °C for 2 days in the dark. After the pretreatment, the seeds are transferred to a germination environment at 35 °C for an additional 2 days. The seeds and seedlings are pulverized into a fine powder using liquid nitrogen and kept at −80 °C until analysis. To release MDA, pulverized tissue was combined with a trichloroacetic acid-based extraction buffer. Under acidic circumstances, thiobarbituric acid was mixed with extracted materials to produce a colorful complex with MDA. After that, a spectrophotometer measures color intensity at 532 nm. MDA concentration was calculated by comparing absorbance to a reference curve of known amounts [28]. The measurement of CAT activity was conducted according to the method established by [29], with minimal modifications. 1 g of the material was ground in 10 mL of 0.1 mol/L sodium phosphate buffer (pH 7.5, 40 g/L PVP, and 5 mmol/L DTT) in an ice bath. After a 20-min centrifugation at 6000 × g at 4 °C, 100 μL of the supernatant was mixed with 2.9 mL of 20 mmol/L H_2_O_2_ solution and acclimatized for 5 min at 25 °C. A unit of CAT activity is a 0.01 absorbance decrease at 240 nm per gram of barley sample per minute (U/g DW).

#### 2.6.3 GABA-T activity assay

The activity of GABA-T was assessed according to the methodology outlined by [30]. To achieve this, a crude enzyme extract was incorporated into a phosphate buffer containing NAD and β-mercaptoethanol. α-ketoglutarate and GABA 1:1, 100 µl were introduced, and the mixture was incubated at 30 °C for 30 min. Absorbance was quantified at 340 nm via a UV–VIS spectrophotometer.

#### 2.6.4 GAD activity assay

A GAD (Glutamic Acid Decarboxylase) activity assay measures the enzymatic conversion of glutamate to gamma-aminobutyric acid (GABA). The activity of GAD was determined according to the method of Bai et al. [29] with some modifications. One gram sample were mixed with 6 mL of ice-cold PBS buffer (70 mM, pH 5.8, containing 0.2 mM PLP, 2 mM β-mercaptoethanol, 2 mM EDTA and 10 % glycerol) and pestled to a slurry at 4 °C. The homogenate was centrifuged at 12,000 × g for 30 min at 4 °C, and the supernatant was used for the enzyme assay. One unit of GAD activity was equal to a release of 1 μmol of GABA produced from glutamate per hour at 40 °C, and expressed as U/g FW.

#### 2.6.5 Determination of xanthine oxidase (XOD) inhibitory activity

An assessment was conducted on the inhibitory efficacy of specific varieties of ZQ2000 and XMLY22 barley treatments (TFUT, germinated (G), TFUT + G, and traditionally soaked (TS) in terms of xanthine oxidase activity. In brief, the following suspension was prepared: 0.3 mM xanthine (22.8 mg was combined with 500 µL 1 M NaOH to create a final concentration of 0.3 mM), Tris-HCL buffer (0.05 mM, pH 7.40), and a positive control (allopurinol) or a range of sample quantities (0.125–2000 µg/ml). An equal volume of Tris-HCL buffer (0.05 mM, pH 7.40), xanthine oxidase (0.11 U/ml in Tris-HCL buffer), and sample were added to the 96-well plates. After adding 150 µL of xanthine and keeping it at 37 °C for 8 min, the reaction was started. The absorbance at 292 nm was monitored using Molecular Devices’ SpectraMax i3 instrument, Silicon Valley, CA, US. Allopurinol was used as a positive control and Tris-HCL buffer as a negative control [31].

The XOD inhibitory activity was evaluated by using the formula = a-b/a*100.

#### 2.6.6 Analysis of *α*-amylase inhibitory activity

To determine the activity of α-amylase, an enzyme solution (50  μL, 10 U mL − 1) was combined with 100  μL of 20  mmol dm^−3^ phosphate buffer (pH 6.9). The mixture was then heated at 37 °C for 10  min before being added to 100  μL of 0.5 % starch solution. The mixture was then incubated at 37 °C for 30  min. Afterward, 100 μL of DNS solution was added prior to incubation at 100 °C for 10 min. A microplate reader (Tecan Spark, Männedorf, Switzerland) was employed to measure the absorbance at 540  nm. The inhibition percentage was determined per the following formula [32].

$$Inhibition\left(\%\right)=1-B-bA-a\times 100$$

where A represents the absorbance intensity of the reaction mixture that contains enzyme solution but lacks CLE, a represents the absorbance intensity of the reaction mixture that lacks enzyme solution and CLE, B represents the absorbance intensity of the reaction mixture that contains enzyme solution and CLE, and b represents the absorbance intensity of the reaction mixture that contains CLE but lacks enzyme solution. Triplicate measurements were conducted on each sample.

#### 2.6.7 Analysis of *α*-glucosidase inhibitory activity

The *α*-glucosidase activity was analyzed; briefly, 50  μL of enzyme solution (0.2 U mL^−1^) was combined with 50  μL of CLE or 25  mmol dm^−3^ phosphate buffer (pH 6.9), and the mixture was incubated at 37 °C for 10  min. The reaction mixture was incubated at 37 °C for 30 min after adding 100 μL of (4-Nitrophenyl β-D-glucopyranoside) PNPG solution (1 mg/mL). The reaction was subsequently terminated with 100 μL of 0.02 mol/dm3 sodium carbonate solution. The IC_50_ of CLE to *α*-glucosidase was calculated in the same manner as previously described herein, and the absorbance at 405 nm was recorded. Triplicate measurements were conducted on all samples [32].

### 2.7 Determination of *γ*- aminobutyric acid (GABA) content

The content of GABA in differently treated barley samples were quantified as described in our earlier study [33], [34], [35]. For this purpose take1g barley flour, 1:10 vol ratio added 70 % Ethanol, vortex to mix1min, at room temperature (25 °C) Ultrasonic extraction 30 min, on 5000 rpm centrifuge 10 min, transfer the supernatant into 25 mL In the volumetric flask, the sample residue is reused 10 mL The extraction solution is extracted once, Combine the two extracts and adjust the volume to 25 mL, shake well and wait for derivatization. Derivatization reaction: Take 1 ml of supernatant and add 0.2 ml 0.04g/ml NaHCO3,0.4 ml4-Dimethylaminoazobenzene-4-sulfonyl chloride, vortex to mix, at 70Incubate at ℃ 20 min. Cool to room temperature, use a disposable needle to draw the reaction solution, and pass it through the membrane (0.22um), load 1.5 ml liquid phase injection bottle to be tested. (λ = 436 nm, column temperature 30 °C, injection volume 10ul, flow rate 1 ml/min) (isocratic elution). The chromatographic separation was carried out on an HPLC (Agilent 1200, USA) provided with an ultraviolet detector. The analytical column was a 5 μm C18 column (150 mm × 4.6 mm). The mobile phase A 50 mM/L Sodium acetate buffer; mobile phase B: Acetonitrile the allowed time of separation of GABA was within 20 min at a constant temperature of 30 °C with 1.0 mL/min flow rate.

### 2.8 Gene expression analysis by real-time RT-PCR

Liquid nitrogen was used to grind barley seeds into a fine powder, and the RNA mini reagent (Life Technologies) was used to isolate total RNA. The ReverTra Ace qPCR RT Master Mix (Toyobo) synthesized first-strand cDNA following the manufacturer’s instructions. PAL, C4H, 4CL, CHS, F3H, C3H, HCT, and FLS gene expression levels were assessed using frozen barley powder samples. After 40 amplification cycles, primer-amplified specificity was verified using melting-curve analysis. The CFX96 Real-Time PCR detection system (Bio-Rad) was employed to conduct quantitative real-time PCR with the SYBR Green PCR Master Mix (Bio-Rad) and a 10-fold cDNA dilution of the template. Initially, the PCR was conducted at 95 °C for 3 min, followed by 40 cycles of 20 sec at 95 °C, 20 sec at 58 °C, and 30 sec at 72 °C. 5.8S rRNA was chosen as the reference gene, and the 2^−ΔΔCt^ method was employed to calculate relative gene expression. The data was analyzed using Biorad-iQ5 software and depicted as a fold expression change relative to the control group [36].

### 2.9 Fourier transform infrared spectroscope (FTIR)

The finely ground samples of ZQ2000 and XMLY22 barley treatments (TFUT, germinated (G), TFUT + G, and traditionally soaked (S)) were placed on the console with an ATR-FTIR spectrometer germanium crystal (Thermo Nicolet Co., Nicolet iS50, USA). Spectra were acquired across 32 scans with a 4 cm^−1^ spectral resolution using an attenuated total reflectance (ATR) accessory, covering wavelengths from 4000 to 500 cm^−1^. Analysis and processing of spectra were done using OMNIC 9.0 (Thermo Fisher Scientific, Waltham, MA, USA). Further, the composition of the protein secondary structure was determined using PeakFit v4.12 software [37].

### 2.10 Scanning electron microscope analysis

The morphological changes in the optimized ZQ2000 barley variety treatments (TFUT, germinated (G), TFUT + G, and traditionally soaked (S)) were examined using a scanning electron microscope (S-3400 N; Hitachi High Technologies, Tokyo, Japan). The samples were coated with gold using a gold-plating instrument and were then examined using a SEM system (SU8000TMP, Hitachi, Japan) at a 10 kV acceleration voltage and image magnifications of 200 × .

### 2.11 Molecular docking

The molecular docking technique was conducted *via* the educational version of MOE software, 2015, USA. The molecular interactions were performed between the structure of ferulic acid (FA; PubChem CID: 445858) as ligand and each of peroxidase (PDB ID: 1APX), polyphenol oxidase (PDB ID: 5CE9), xanthine oxidase (PDB ID: 1FIQ), α-amylase (PDB ID: 1OSE), and α-glycosidase (PDB ID: 1UOK) enzymes as macromolecule receptor which were retrieved protein data bank (https://www.rcsb.org/) and PubChem Database (https://pubchem.ncbi.nlm.nih.gov/). Each structure was then optimized by combining fractional charges, and energy was minimized using Protonate-3D and MMFF94X force fields, and H_2_O was removed, structure refining, energy minimization, and 3D protonation *via* MOE. The process was repeated for the composites, and thereafter 4–5 appropriate docked postures were created which were visualized and analyzed then for their hydrophobicity, electrostatics potential, H-bonds, and heat-map structure fluctuations *via* heatmapper (http://heatmapper.ca/expression/) [38], [39].

### 2.12 Statistical analysis

The data obtained for each parameter was subjected to statistical analysis using Statistical Package Origin-Pro 8.5. The experiment was performed with a completely randomized design (CRD) and standard deviation, and the analysis of variance was applied to determine the level of significance followed by LSD. All the experiments were performed in triplicate.

## 3 Results and discussion

### 3.1 Effect of different treatments on TPC and TFC content of barely

**Fig. 1:**
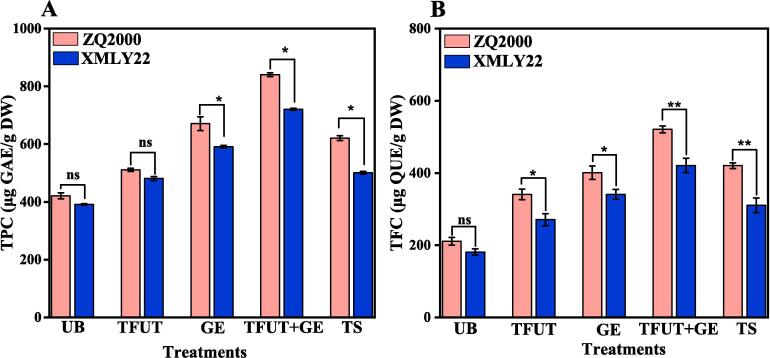
Illustrates the effects of untreated barley (UB), triple-frequency ultrasound treatment (TUFT), germination (GE), the combined effect of TFUT + GE and traditional soaking (TS) on the TPC (A) and TFC(B) of two barley varieties, including ZQ2000 and XMLY22.

The study examined the effects of different treatments, including untreated barleys (UB), triple-frequency ultrasound treatment (TFUT), germinated barleys (GE), combined effect of TFUT + GE, and traditional soaking (TS), on the TPC and TFC of barley varieties (ZQ2000 and XMLY22). All samples treated with diverse treatments showed a significant (p < 0.05) increase in TPC and TFC when compared to the control group, as illustrated in Fig. 1. The concentrations of TPC and TFC were significantly increased following exposure to TFUT, GE, TFUT + GE, and TS in ZQ2000 (TPC: 21.44, 59.51, 99.90, and 47.59 %; TFC: 61.69, 90.16, 147.13, 99.46 %) and XMLY22 (TPC: 23.05, 51.13, 84.29, 28.10 %; TFC: 49.55, 88.29, 132.55, 71.71 %). The TPC increased from 420.56 μg of GAE/g DW (UB) to 720.33 μg GAE/g DW (TFUT + GE) and from 210.73 μg of GAE/g DW (UB) to 520.79 μg of GAE/g DW (TFUT + GE) for the ZQ2000 and XMLY22 barley varieties, respectively (Fig. 1A). Comparable patterns of the TPC in samples subjected to ultrasound treatment have been documented in tofu whey, strawberry juice, and lime juice [40], [41]. These results indicate that ultrasound enhances seed germination, while ultrasonic-induced cavitation and chemical effects modify the cellular structure of seeds, facilitating the release of bioactive chemicals and thereby elevating the phenolic content [42], [43].

Fig. 1B also presents the results regarding the effects of different treatments (UB, TFUT, GE, TFUT + GE, and TS) on the TFC of barley varieties (ZQ2000 and XMLY22). The TFC of both varieties also showed a significant increase in treated samples when compared to the untreated barley samples (UB), as observed for the TPC. The TFC increased from 210.73 μg of GAE/g DW (UB) to 520.79 μg GAE/g DW (TFUT + GE) and from 180.96 μg of GAE/g DW (UB) to 420.84 μg of GAE/g DW (TFUT + GE) for the ZQ2000 and XMLY22 barley varieties, respectively. The highest TFC measured, as shown by ZQ2000 (TFUT + GE), was 520.79 μg of GAE/g DW. Similar results were reported in an earlier study where grapefruit and apple juice treated with ultrasound showed a discernible, substantial increase in total flavonoids [44]. The increase in TPC and TFC may also be attributed to the action of free radicals, which may initiate the synthesis of phenylalanine ammonia-lyase as a stress signal, thereby increasing the synthesis of polyphenol flavonoid and other substances [45].

### 3.2 Effects of different treatments on individual phenolic acids and flavonoids content of barley

Table 1 illustrates the impact of diverse treatments, namely TFUT, GE, TFUT + GE, and TS, on the phytochemical content of barley varieties (ZQ2000 and XMLY22), encompassing gallic acid, ferulic acid, caffeic acid, p-coumaric acid, chlorogenic acid, catechin, *iso*-quercitrin, rutin, and kaempferol. The results indicated that TFUT increases the content of gallic acid (ZQ2000: 3.05 to 5.18 μg/g of DW, XMLY22: 2.19 to 3.73 μg/g of DW), chlorogenic acid (ZQ2000: 20.15 to 32.56 μg/g of DW, XMLY22: 18.15 to 30.23 μg/g of DW), and rutin (ZQ2000: 27.89 to 34.56 μg/g of DW, and XMLY22: 21.16 to 29.67 μg/g of DW) whereas decreases the content of ferulic acid (ZQ2000: 220.27 to 180.36 μg/g of DW, and XMLY22: 200.29 to 160.65 μg/g of DW), caffeic acid (ZQ2000: 35.16 to 31.25 μg/g of DW, and XMLY22: 20.19 to 16.24 μg/g of DW), p-coumaric acid (12.10 to 7.14 μg/g of DW, and XMLY22: 7.10 to 5.23 μg/g of DW), and kaempferol (ZQ2000: 27.89 to 34.56 ± 0.65 μg/g of DW, and XMLY22: 21.16 to 29.67 μg/g of DW) of barely seeds. In contrast, the phenolic acids and flavonoid content in the seeds of both barley types exhibited a significant increase relative to the control samples after undergoing germination, immersion, and TFUT + GE combined treatment. The biological activity of the examined samples is significantly affected by their variety and growth circumstances [46]. A prior study indicated that abiotic treatment enhanced antioxidant activity and promoted the production of polyphenols and flavonoids with antioxidant characteristics. It has been established that plants generate reactive oxygen species, activate enzyme genes, and synthesize these chemicals in reaction to unfavorable circumstances such as low temperatures, salinity stress, and ultrasonic exposure. Despite drought circumstances, radiation seemed to enhance the antioxidant activity of barley types (Nokia and KWS Olof) [47]. Our findings suggest that integrating several techniques may yield optimal results in relation to health-promoting phytochemicals.

**Table 1:** Effect of ultrasound, germination, and soaking treatments on phenolic acids and (B) flavonoids of two barley varieties, ZQ2000 and XMLY22.

Barley varieties	Treatments	Phenolic acids (μg/g of DW)						Flavonoids (μg/g of DW)			
		Gallic acid	Ferulic acid	Caffeic acid	*p*-coumaric acid	Chlorogenic acid	Catechin	Quercitrin	*iso*-quercitrin	Rutin	Kaempferol
ZQ2000	UB	3.05 ± 0.02^cEFG^	220.27 ± 3.30 ^dDE^	35.16 ± 1.50 ^cBC^	12.10 ± 0.88^c^	20.15 ± 0.98^c^	19.65 ± 0.22 ^d^	15.23 ± 0.99^c^	18.52 ± 0.14^c^	27.89 ± 0.85 ^d^	4.05 ± 0.09 ^d^
	TFUT	5.18 ± 0.03^bC^	180.36 ± 5.24 ^eG^	31.25 ± 1.25 ^cBCD^	7.14 ± 0.78 ^d^	32.56 ± 0.65^b^	17.23 ± 0.23 ^d^	22.50 ± 0.45^b^	15.69 ± 0.65^c^	34.56 ± 0.65^c^	8.13 ± 0.03^b^
	GE	8.26 ± 0.08 ^aAB^	294.56 ± 6.33 ^bABC^	42.15 ± 1.23 ^bB^	20.19 ± 0.98^b^	40.15 ± 0.75 ^a^	28.69 ± 0.65^b^	29.34 ± 0.65 ^a^	26.33 ± 0.23^b^	39.18 ± 0.24^b^	11.24 ± 0.18^b^
	TFUT + GE	10.06 ± 0.02^aA^	370.59 ± 7.56^aA^	52.34 ± 2.20 ^aA^	26.24 ± 0.25 ^a^	46.18 ± 0.88 ^a^	36.73 ± 0.19 ^a^	33.75 ± 0.78 ^a^	33.45 ± 0.22 ^a^	48.59 ± 0.26 ^a^	15.14 ± 0.24 ^a^
	TS	4.23 ± 0.02 ^bD^	250.23 ± 8.56^cD^	38.56 ± 1.98 ^bBC^	15.65 ± 0.19^c^	28.79 ± 0.69^b^	22.95 ± 0.22^c^	26.89 ± 0.26^b^	23.14 ± 0.25^b^	31.47 ± 0.24^c^	6.21 ± 0.65^c^
XMLY22	UB	2.19 ± 0.05^cH^	200.29 ± 2.89 ^bDEF^	20.19 ± 0.98 ^bF^	7.10 ± 0.75^c^	18.15 ± 0.84 ^d^	25.73 ± 0.28^c^	11.12 ± 0.24^c^	14.70 ± 0.19^c^	21.16 ± 0.12 ^d^	7.25 ± 0.25^c^
	TFUT	3.73 ± 0.05 ^bE^	160.65 ± 6.55^c^	16.24 ± 0.65 ^cFG^	5.23 ± 0.25 ^d^	30.23 ± 0.60^b^	20.43 ± 0.45^c^	16.73 ± 0.22^b^	9.50 ± 0.16 ^d^	29.67 ± 0.26^c^	10.14 ± 0.20^b^
	GE	4.56 ± 0.03^aCD^	298.65 ± 8.10 ^aABC^	29.36 ± 0.99 ^aBCD^	12.11 ± 0.65^b^	33.49 ± 0.55^b^	31.45 ± 0.23^b^	24.58 ± 0.15 ^a^	20.21 ± 0.15^b^	35.14 ± 0.83^b^	16.19 ± 0.19 ^a^
	TFUT + GE	5.73 ± 0.06^aC^	330.29 ± 7.23 ^aAB^	35.16 ± 1.75 ^aBC^	19.12 ± 0.68 ^a^	39.73 ± 0.24 ^a^	37.96 ± 0.67 ^a^	29.74 ± 0.10 ^a^	26.28 ± 0.22 ^a^	43.25 ± 0.17^a^	19.09 ± 0.21 ^a^
	TS	3.25 ± 0.09^bEF^	118.54 ± 6.56 ^dG^	23.14 ± 1.23 ^bBCDE^	10.09 ± 0.98^c^	26.23 ± 0.24^c^	29.46 ± 0.85^b^	19.87 ± 0.26^b^	17.45 ± 0.11^b^	25.28 ± 0.23^c^	9.22 ± 0.23^b^

*Different letters in the same column in the table indicate significant differences (p < 0.05). Untreated barley (UB), triple-frequency ultrasound treatment (TUFT), germination (GE), the combined effect of TFUT + GE and traditional soaking (TS).

### 3.3 Antioxidant activity

**Fig. 2:**
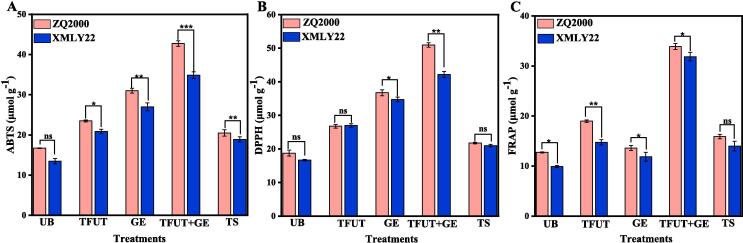
Effects of untreated barley (UB), triple-frequency ultrasound treatment (TUFT), germination (GE), and the combined effect of TFUT + GE and traditional soaking (TS) on the scavenging capabilities ABTS (A), DPPH (B), and FRAP (C) of two barley varieties, including ZQ2000 and XMLY22.

The antioxidant activities of both barley varieties, ZQ2000 and XMLY22, were evaluated through three different assays: ABTS, DPPH, and FRAP. The exposure to TFUT, GE, TFUT + GE, and TS induced an overall increase in antioxidant capacity, as indicated by DPPH, ABTS, and FRAP measurements (Fig. 2). Among all treatments, combined treatments of TFUT and GE (TFUT + GE) presented greater values for ABTS, DPPH, and FRAP. The ABTS increased from 16.71 μmol g^−1^ (UB) to 42.73 μmol g^−1^ (TFUT + GE) and from 13.46 μmol g^−1^ (UB) to 34.87 μmol g^−1^ (TFUT + GE) for the ZQ2000 and XMLY22 barley varieties, respectively (Fig. 2A). The DPPH levels exhibited an increase from 18.73 μmol g^−1^ (UB) to 50.98 μmol g^−1^ (TFUT + GE) and from 16.64 μmol g^−1^ (UB) to 42.17 μmol g^−1^ (TFUT + GE) for the ZQ2000 and XMLY22 barley varieties, respectively (Fig. 2B). The FRAP showcased an increase from 12.69 μmol g^−1^ (UB) to 33.89 μmol g-1 (TFUT + GE) and from 9.89 μmol g-1 (UB) to 31.86 μmol g^−1^ (TFUT + GE) for the ZQ2000 and XMLY22 barley varieties, respectively (Fig. 2C). However, the combination treatment (TFUT + GE) increased the scavenging capabilities of DPPH (ZQ2000: 172.18 % and XMLY22: 159.06), ABTS (ZQ2000: 155.86 % and XMLY22: 222.14), and FRAP (ZQ2000: 166.22 and XMLY22: 222.14) compared to untreated barley (UB). The study observed an increase in antioxidant activity corresponding with the rise in unbound polyphenols and flavonoid contents during the germination process. Another study indicated that the overall levels of free flavonoids, free polyphenols, and total phenols progressively increased throughout the germination process [48]. The antioxidant capacity of polyphenols and flavonoids depends on their concentration and composition, as demonstrated by research [49]. The findings of this study align with previous research indicating that germination can enhance the antioxidant capacity and total phenolic content of mung bean seeds [50]. As a result, we further characterized the composition of barley varieties, ZQ2000 and XMLY22, using untargeted metabolomics technology, which was further enhanced by exogenous GABA in conjunction with ultrasonic treatment.

### 3.4 Effect of different treatments on SOD, POD, MDA, CAT, H_2_O_2_, and PPO content in barley

**Fig. 3:**
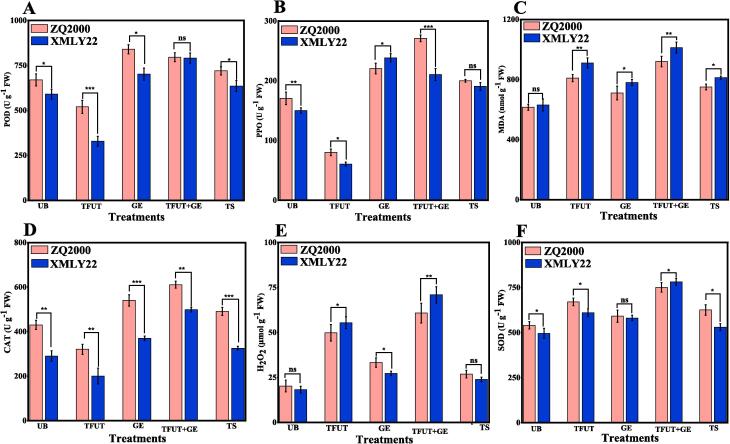
Effects of untreated barley (UB), triple-frequency ultrasound treatment (TUFT), germination (GE), and the combined effect of TFUT + GE and traditional soaking (TS) on (A) POD, (B) PPO, (C) MDA, (D) CAT, (E) H_2_ O_2_ , (F) SOD of two barley varieties, ZQ2000 and XMLY22.

Essential biomarkers and enzymes in oxidative stress and antioxidant defense systems include SOD, POD, MDA, CAT, H2O2, and PPO. POD lowers peroxides and catalyzes the oxidation of phenolic compounds, which increases plant resilience, whereas CAT transforms superoxide radicals and MDA signals lipid peroxidation [51]. The findings of POD and PPO activities subsequent to exposure to TFUT, GE, TFUT + GE, and TS treatments on both barley varieties, including ZQ2000 and XMLY22, are illustrated Fig. 3. The TFUT treatment yielded POD data indicating a decrease from 670.25 U g^−1^ FW (UB) to 520.14 U g^−1^ FW (TFUT) for the ZQ2000 barley variety and from 590.16 U g^−1^ FW (UB) to 328.85 U g^−1^ FW (TFUT) for the XMLY22 barley variety Fig. 3A. Conversely, other treatments (GE, TFUT + GE, and TS) resulted in an increase in POD for both barley varieties. Similarly, a trend was reported in PPO results from the TFUT treatment, which showed that the ZQ2000 barley variety decreased from 170.45 U g-1 FW (UB) to 80.23 U g-1 FW (TFUT), while the XMLY22 barley variety decreased from 150.23.16 U g-1 FW (UB) to 60.55 U g-1 FW (TFUT) (Fig. 3B). Ultrasonication can either activate or inactivate enzymes, depending on the enzymes**’** nature, the ultrasonic conditions, and the types of samples. The mechanical and chemical effects produced by ultrasound cavitation were responsible for the inactivation of PPO and POD activity. A comparable trend was observed in earlier investigations, where ultrasonication led to a reduction in the PPO activity of quince juice, attributed to the disruption of the tertiary structure and the loss of the α-helix [52].

As a result, the distinct effects of ultrasonic treatment on PPO and POD may lead to an increase in enzyme activities, as evidenced by the desirable conformational changes of the enzyme [53] or the up-regulation of enzyme production in living plant cells [54]. In contrast, the secondary and tertiary structures of enzymes are disrupted by certain other ultrasonic conditions, which produces an increased mechanical force that leads to the inactivation of PPO and POD [54]. The catalyst of NADPH oxidase and the respiratory action are responsible for the production of O2.-, H_2_O_2_, and OH.- by ROS sources. The toxicity that elevated ROS levels pose to plants underscores the critical role of detoxifying enzyme activities in maintaining cellular homeostasis of ROS levels [55]. In the ROS system, O_2_.- is initially elevated in response to the stress and is swiftly converted to H_2_O_2_ through the catalysis of SOD. As a result, the removal of H_2_O_2_ is achieved by employing CAT and POD. The current study evaluated the H_2_O_2_, MDA, CAT, and SOD activities in response to a variety of interventions. As can be seen in Fig. 3C, the TFUT + GE treatment (49.60, and 60.53 %) exhibited a higher MDA content than the TFUT (31.70, and 44.40 %), GE (15.44, and 23.73 %), and TS (22.00, and 28.81 %) treatments. Furthermore, the control group’s CAT activity increased from 430.25 U g^−1^ (ZQ2000) and 290.33 U g^−1^ (XMLY22) to peak levels of 610.59 U g^−1^ (ZQ2000) and 498 U g^−1^ (XMLY22) subsequent to the application of TFUT + GE treatment. In comparison to UB, GE, TFUT + GE, and TS groups, TFUT treatment exhibited decreased the CAT activities (Fig. 3D). Fig. 3E illustrate that the H_2_O_2_ content increased following exposure to TFUT, GE, TFUT + GE, and TS treatments. The values for ZQ2000 were 146.55, 64.83, 201.08, and 33.18 %, while those for XMLY22 were 204.56, 49.61, 289.50, and 31.26 %. Furthermore, the SOD activity in the control samples (UB) exhibited an increase from 540.18 U g^−1^ (ZQ2000) and 495.87 U g^−1^ (XMLY22) to peak levels of 750.68 U g^−1^ (ZQ2000) and 780.69 U g^−1^ (XMLY22) subsequent to the application of the TFUT + GE treatment (Fig. 3E). These enzymes catalyze the oxidation of phenolic compounds when hydrogen peroxide is present or under aerobic conditions, resulting in the discoloration of products. The disruption of cell structure induced by ultrasonication may facilitate the oxidation of phenolic compounds, thereby affecting the color, taste, nutritional profile, and bioactive properties of plant-based foods.

### 3.5 Effects of different treatments on GABA, GAD, and GABA-t contents in barely seeds

**Fig. 4:**
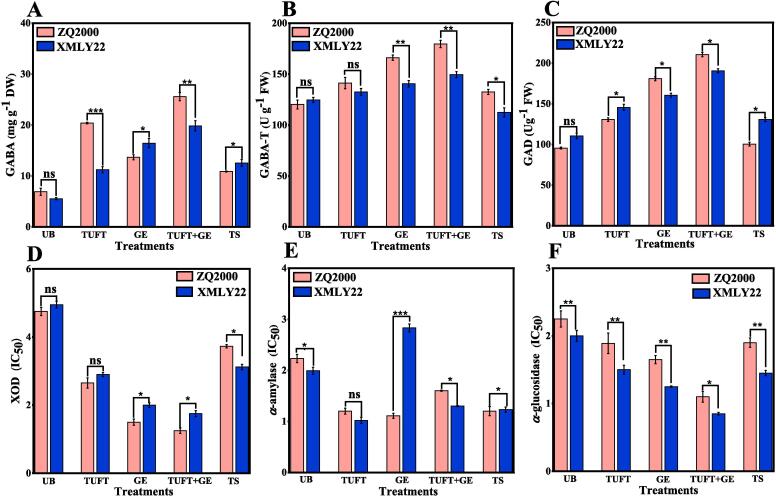
Effects of untreated barley (UB), triple-frequency ultrasound treatment (TUFT), germination (GE), and the combined effect of TFUT + GE and traditional soaking (TS) on (A) GABA, (B) GABA-T, (C) GAD, (D) XOD, (E) α-amylase, (F) α-glucosidase of two barley varieties, ZQ2000 and XMLY22.

GABA, a natural nonprotein amino acid that is universally present in microorganisms, animals, and plants, is synthesized through the GABA shunt and the polyamine degradation pathways. Glutamate decarboxylase (GAD) is the rate-limiting enzyme in the GABA shunt pathway, as it converts glutamate into GABA [56]. GABA is synthesized in the polyamine degradation pathway by diamine oxidase (DAO), polyamine oxidase (PAO), and γ-aminoaldehyde dehydrogenase (AMADH), with DAO functioning as the primary enzyme. The synthesized GABA is subsequently catabolized by γ-aminobutyric acid transaminase (GABA-T, EC 2.6.1.19) and then participates in the tricarboxylic acid (TCA) cycle [57]. In the present investigation, we examined the influence of TFUT, GE, TS, and TUFT + GE on the accumulation of GABA content in both barley varieties ZQ2000 and XMLY22. The GABA content levels in ZQ2000 and XMLY22 exhibit notable increases (2.9, 1.98, 3.70, and 1.57-fold) and 2.02, 2.96, 3.58, and 1.54-fold) in comparison to the control (UB) when treated with TUFT, GE, TUFT + GE, and TS, respectively (Fig. 4A). GABA is synthesized in plant cells through the catalysis of GAD enzyme from glutamic acid. Subsequently, GABA-T enzyme degrades it, allowing it to participate in the tricarboxylic acid cycle. Consequently, the metabolism of GABA is contingent upon the activity of the GAD enzyme and the GABA-T enzyme [32]. After TUFT, GE, TUFT + GE, and TS treatments, GABA-T activities increased significantly (p < 0.05) to 1.17, 1.38, 1.49, and 1.10-fold (ZQ2000), and 1.06, 1.12, 1.19, and 0.90-fold (XMLY22), compared to the control sample (UB) (Fig. 4B). Similarly, the GAD activities were substantially (p < 0.05) increased to 1.36, 1.27, 1.83, and 1.05-fold (ZQ2000) and 1.31, 1.10, 1.72, and 1.18-fold (XMLY22) following TUFT, GE, TUFT + GE, and TS treatments compared to the control sample (UB), respectively (Fig. 4C). A previous study documented that GAD activity is stimulated during germination, leading to the conversion of GLU to GABA. The GAD enzyme catalyzes the conversion of Glu to GABA, which is then converted to SSA and succinic acid by the GABA-T enzyme [58]. Consequently, TFUT, GE, and TS treatments may increase the activity of the GAD enzyme, resulting in a decrease in Glu content and an increase in GAD activity. The enrichment of GABA in barely seeds under TFUT, GE, and TS treatments may be attributed to increased cell membrane permeability, increased substrate concentration, and accelerated reactions. Additionally, ultrasonic waves had a substantial impact on the biosynthesis and metabolism of GABA, leading to its increased accumulation [59].

### 3.6 Effect of different treatments on alpha-amylase, alpha-glucosidase, and xanthine oxidase inhibitory activities of barely

α-amylase and α-glucosidase, also known as maltase, are enzymes that break down starch, a primary source of carbohydrates for mammals, and can regulate postprandial blood sugar levels [60]. The combined treatment (TFUT + GE) of ZQ2000 (1.02 mg/ml) and XMLY22 (1.20 mg/ml) exhibited higher IC_50_ values of α-amylase than the control treatments (2.23 mg/ml and 2.25 mg/ml) (Fig. 4E). Similarly, the combined treatment (TFUT + GE) of ZQ2000 (1.1 mg/ml) and XMLY22 (0.85 mg/ml) demonstrated greater IC_50_ values of α-glucosidase compared to the control treatments (2.83 mg/ml and 2.00 mg/ml) (Fig. 4F). Molybdenum, a component of xanthine oxidase (XOD), converts hypoxanthine to xanthine and xanthine to uric acid (UA). The inflammatory pathway is influenced by UA deposition due to XOD overactivity, associated with reactive oxygen species. To reduce UA levels, inhibiting UA synthesis through XOD inhibition is the initial treatment [61]. Fig. 4D presents the findings regarding the XOD inhibitory activity observed in the barley varieties ZQ2000 and XMLY22. The TFUT + GE treatment exhibited the most pronounced XOD inhibitory activity, with measurements of 1.25 mg ml^−1^ for ZQ2000 and 1.75 mg ml^−1^ for XMLY22. In contrast, the TFUT sample recorded values of 2.65 mg ml^−1^ for ZQ2000 and 2.9 mg ml^−1^ for XMLY22, while the GE treatment showed 1.50 mg ml^−1^ for ZQ2000 and 2.0 mg ml^−1^ for XMLY22. The TS sample had values of 3.73 mg ml^−1^ for ZQ2000 and 3.12 mg ml^−1^ for XMLY22, and the UB sample recorded 4.75 mg ml^−1^ for ZQ2000 and 4.95 mg ml^−1^ for XMLY22. The concentration of polyphenolic compounds is strongly connected with the strong inhibitory activity of several treated barley seeds against clinically significant enzymes, including α-amylase, α-glucosidase, and XOD. As mentioned previously, the concentrations of various secondary metabolites, including gallic acid, ferulic acid, caffeic acid, p-coumaric acid, chlorogenic acid, catechin, quercitrin, *iso*-quercitrin, rutin, and kaempferol, are increased in TFUT, GE, TFUT + GE, and TS samples. These polyphenolic compounds are closely linked to the inhibition of α-amylase, α-glucosidase, and XOD due to their unique characteristics, such as planar structure, hydrophobicity, binding mechanism, and hydroxyl group. These characteristics allow them to impede the formation of blood glucose and uric acid by binding to enzymes [32], [61]. Overall, ultrasonic treatment (TFUT) significantly decreased XOD activity in both ZQ2000 and XMLY22 compared to the untreated barley (UB). The findings suggest that the elevated levels of α-amylase, α-glucosidase, and XOD inhibitory activities in barley may be ascribed to the augmented secondary metabolites, such as gallic acid, ferulic acid, caffeic acid, p-coumaric acid, chlorogenic acid, catechin, quercitrin, *iso*-quercitrin, rutin, and kaempferol [59], [61].

### 3.7 Relative expression of polyphenolic compounds and their metabolism genes

Polyphenols, flavonoids, and other antioxidants that protect plants from stress are produced via the phenylpropanoid metabolic pathway [3]. This metabolic process begins with phenylalanine, which is metabolized to *trans*-cinnamic acid by PAL. Cinnamic acid 4-hydroxylase catalyzes *trans*-cinnamic acid to produce coumaric acid, ferulic acid, caffeic acid, and other chemical compounds. Finally, 4-coumarate-CoA ligase (4CL), cinnamyl-alcohol dehydrogenase (CAD), caffeate 3-O-methyltransferase (COMT), CCoAOMT, and CCR facilitate the synthesis of phenylpropanoid metabolites, including lignin, flavones, and polyphenols.

The study aimed to understand the accumulation of polyphenolic compounds in both barley varieties (ZQ2000 and XMLY22) after TFUT, GE, TFUT + GE, and TS by examining the expression of genes encoding the biosynthesis pathway of identified phenolic acids and flavonoid compounds, including PAL, C4H, 4CL, CHS, F3H, C3H, HCT, and FLS.

**Fig. 5:**
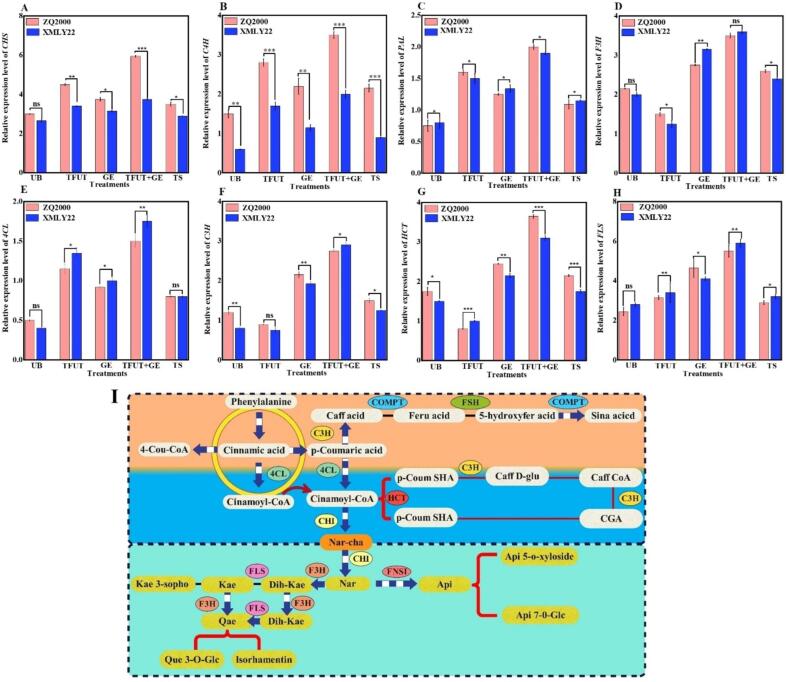
Effects of untreated barley (UB), triple-frequency ultrasound treatment (TUFT), germination (GE), and the combined effect of TFUT + GE and traditional soaking (TS) (A) CHS, (B) C4H, (C) PAL, (D) (F3H), (E) 4CL, (F) C3H, (G) HCT, FLS (H) and biosynthesis pathway of identified polyphenolic compounds (I) of two barley varieties, ZQ2000 and XMLY22. *Abbreviations of metabolites and enzymes: 4-Cou-CoA: 4-coumaroyl-CoA, p-Coum SHA: p-coumaroylshikimic acid, Api: Apigenin, Api 5-O-xyloside: Apigenin 5-O-xyloside, Api 7-O-Glc: Apigenin 7-O-glucoside, p-Cou QA: p-coumaroylquinic acid, Caff D-glu: Caffeoyl D-glucose, Caff CoA: Caffeoyl CoA, caff acid: caffeic acid, feru acid: ferulic acid, 5-hydroxyfer acid: 5-hydroxy ferulic acid, sina acid: sinapic acid, CGA: Chlorogenic acid, Nar-cha: Naringenin chalcone, Nar: Naringenin, Dih-Kae: dihydrokaempferol, Kae: Kaempferol, Kae 3-sopho: Kaempferol 3-sophorotrioside, Dih-Que: dihydroquercetin, Que: Quercetin, Que 3-O-Glc: Quercetin 3-O-glucosyl-rhamnosyl-glucoside, PAL: Phenylalanine ammonia-lyase, C4H: cinnamate-4-hydroxylase, COMT: Catechol-O-methyltransferase, CHI: Chalcone isomerase, CHS: Chalcone synthase, 4CL: 4-coumarate-CoA ligase, CHR: chalcone reductase, F3′H: Flavanone-3-hydroxylase, FLS: flavonol synthase, F5H: ferulate-5-hydroxylase, HCT: hydroxycinnamoyl transferase.

Our findings deliver a deeper insight into the accumulation of polyphenolic compounds following TTFUT, GE, TFUT + GE, and TS (Fig. 5). The expression levels of PAL, C4H, 4CL, CHS, F3H, and FLS were notably increased in the barley variety ZQ2000. The expression levels of C3H and HCT were significantly decreased under TFUT treatment when compared to the control group (p < 0.05). In contrast, the exposure of XMLY22 (a barley variety) to the TFUT resulted in the down-regulation of F3H, C3H, and HCT expression levels while simultaneously leading to the up-regulation of PAL, C4H, 4CL, CHS, and FLS when compared to the control. Simultaneously, the GE, TFUT + GE, and TS treatments led to an increase in the relative expression levels of PAL, C4H, 4CL, CHS, F3H, C3H, HCT, and FLS when compared to the control group. The correlation analysis indicated a robust relationship among the genes associated with polyphenolic compounds, the reactive oxygen species (ROS) system in barley seeds, and the previously mentioned compounds. The results validated the perspective of ROS as precursor signal molecules for the accumulation of GABA in barley seeds and phenolic compounds [4], [52].

### 3.8 FTIR spectroscopy

**Fig. 6:**
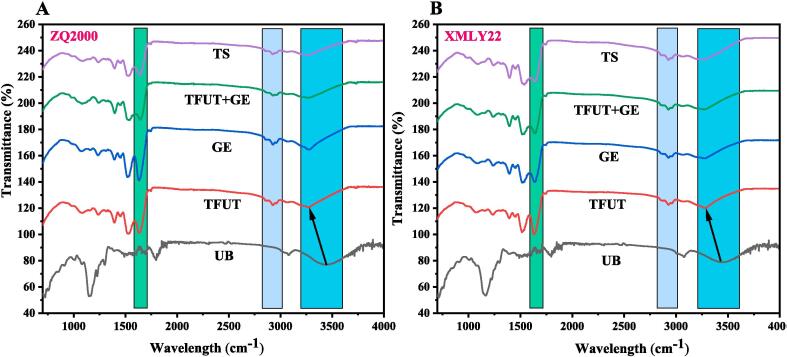
FTIR spectra analysis of two barley varieties, ZQ2000 (A) and XMLY22 (G). Untreated barley (UB), triple-frequency ultrasound treatment (TUFT), germination (GE), and the combined effect of TFUT + GE and traditional soaking (TS).

FTIR spectroscopy analysis was employed to examine the changes in physicochemical parameters of both barley varieties, ZQ2000 and XMLY22, subjected to various treatments. The FTIR spectra of UB, TFUT, GE, TFUT + GE, and TS samples are presented in Fig. 6, which showed that chemical bonds and their presence in the biological material can be revealed by specific distinct groups in the mid-infrared region. Absorption spectra in the 2900–3500 cm^−1^ region indicated hydroxy and/or N-H vibrations. The absence of an absorbance peak in the 2000–2500 cm^−1^ range indicated the absence of chemical groups like C≡N, C≡C, or C≡C≡C clusters. The amide-I band, which corresponds to proteins, was identified at 1600–1700 cm^−1^ due to vibrations related to C≡O bond stretching and out-of-phase vibrations on C-N bond stretching [62]. Additionally, the absorption bands near 2900 cm^−1^ reflected the presence of alkyl CH_2_ groups [63]. UB samples of both varieties (ZQ2000 and XMLY22) exhibited a pronounced band shift from 3448 cm^−1^ to 347 cm^−1^, demonstrating that all treatments (TFUT, GE, TFUT + GE, and TS) exert a significant influence on the barley samples, with distinctive interactions, as illustrated in Fig. A-B. The absorption band at 3080 cm^−1^ and the band shifting from 3077 cm^−1^ to 2924 cm^−1^ indicated the asymmetrical stretching of the C–H bond in CH_3_ and CH_2_, illustrating the effect of all treatments (TFUT, GE, TFUT + GE, and TS).

Additionally, a band at 2852 cm^−1^ and 2854 cm^−1^ in all treatments (TFUT, GE, TFUT + GE, and TS) of ZQ2000 and XMLY22 samples, respectively, with the exception of UB, indicated CH_2_ symmetrical stretching. UB did not show this band, likely because this stretching was converted into other forms. Furthermore, the transition from 956 cm^− 1^ in TFUT + GE sample (XMLY22) to 884 cm^− 1^ indicated a change in NO_2_ stretching or CH_2_ rocking. TFUT-GE exhibits more robust hydrogen bonding compared to other treatments, and there is a more pronounced shift in the O-H absorption peak within the TFUT-GE group than in the GE group. Ultrasonic cavitation induces the formation of hydrogen bonds among the molecules of highland barley starch [64].

The results indicated that the TFUT treatment modified the structure of ZQ2000 and XMLY22, leading to the emergence of new interaction patterns. All these alterations can be ascribed to the breaking of the cell wall carbohydrate chain due to ultrasonic and germination treatment. Significant variations in percentage transmittance in the treated samples can be attributed to carboxylic acids, amines, and esters. The increase in transmittance percentage (%) in the ester group may result from the reduction of polarity and the creation of color. The treatment outcomes of UB, TFUT + GE, TFUT + GE, and TS exhibited significant variations in the percentage transmittance of C = O molecules. Similar FT-IR spectral data analysis was observed in the PEF and US-treated extracts of almonds [65] and apple seed proteins [66]. The FTIR spectroscopy results align with the SEM pictures, indicating that sonication can expedite the cleavage of glycosidic connections between monosaccharide units, leading to the degradation of cell wall structure.

**Fig. 7:**
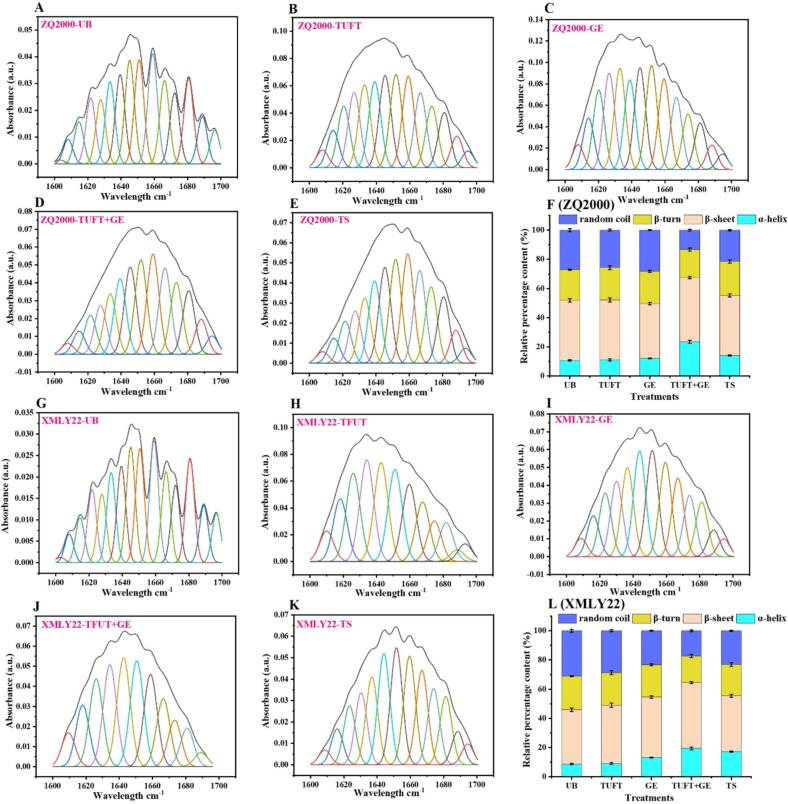
Decomposition (A–E) and relative percentage content (F) of ZQ2000, and decomposition (G–K) and relative percentage content (L) of XMLY22 treated with diverse treatments. Untreated barley (UB), triple-frequency ultrasound treatment (TUFT), germination (GE), and the combined effect of TFUT + GE and traditional soaking (TS).

The secondary structure of both barley varieties, subjected to various treatments, including UB, TFUT, GE, TFUT + GE, and TS samples, was characterized and analyzed using OMNIC and peakfitTM Software after data transformation and deconvolution of amid-I at wavenumbers 1600 to 1700 cm^− 1^. After undergoing various treatments, it was found that the secondary structures of both barley varieties, XMLY22 and ZQ2000, were modified significantly (Fig. 7). The ZQ2000 exhibited an increase in the proportion of *α*-helices from 10.81 % (UB) to 23.43 % (TFUT + GE) (Fig. 7F), while the XMLY22 showed an increase in the proportion of *α*-helices from 8.81 % (UB) to 19.43 % (TFUT + GE) (Fig. 7L). Similarly, the percentage of *β*-sheet in the ZQ2000 increased from 41.04 % (UB) to 44.08 % (TFUT + GE) (Fig. 7F) and from 37.04 % (UB) to 45.08 % (TFUT + GE) (Fig. 7L) for the XMLY22.

Conversely, for the ZQ2000 samples, the percentage of *β*-turn dropped from 21.08 % (UB) to 19.16 % (TFUT + GE) (Fig. 7F) and for the XMLY22 samples, from 23.08 (UB) to 18.17 % (TFUT + GE) (Fig. 7L). Furthermore, compared to other treatments, the sample’s (TFUT + GE) random coil composition was observed to be lower. While the *α*-helix, *β*-turn, and random coil forms are more flexible, the *β*-sheet shape is known for its relative stability [62]. The aforementioned changes exhibited that TFUT increased the elasticity and enhanced the secondary structure of barley. The findings revealed that the combined effect of sonication and germination (TFUT + GE) had an effect on the secondary structural composition of GABA as well as other amino acids from other proteins, particularly the *α*-helix and *β*-sheet. The sonication process, which broke or unraveled the connections between various sections of protein molecules and local amino acid sequences, is responsible for the reported results. Several elements contributing to this disruption include microjets, turbulence, shock waves, shear force, and free radicals [62].

### 3.9 Morphological observation

**Fig. 8:**
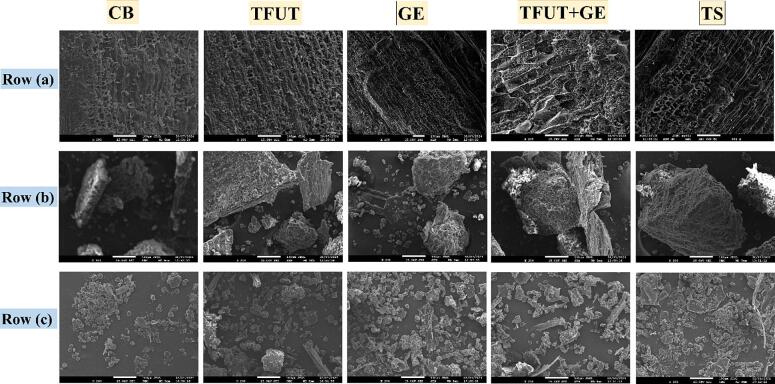
SEM-micrographs of outer layer (Row a), half dissection (Row b), and powder (Row c) of ZQ2000, treated with diverse treatments. Untreated barley (UB); triple-frequency ultrasound treatment (TUFT); germination (GE); and the combined effect of TFUT + GE and traditional soaking (TS).

SEM images were taken to illustrate the alterations in the biological structure of barley, comparing states before and after five distinct treatments: UB, TFUT, GE, TFUT + GE, and TS treatments. The assessment is presented in terms of the outer surface, half dissection and powdered form. The outer layer of the control sample exhibits a compact and orderly structure; however, the application of ultrasonic treatment disturbs the surface, leading to an increase in porosity. The process of germination further compromises the outer sheath, resulting in the formation of elongated structures that exhibit a distinct definition. Soaking fragmentation manifests to a significantly lesser degree compared to the combined effects of TFUT and GE treatment, which leads to the most pronounced breakdown (Fig. 8). In the half dissection images, the control samples exhibited a dense and well-compacted appearance; however, ultrasound has the capacity to fragment particles or induce their separation. Germination further disturbs the outer layer, producing elongated formations with distinct features. Soaking fragmentation happens to a significantly smaller degree than the TFUT + GE treatment, which leads to the most considerable disintegration. The control sample powder exhibits a greater homogeneity and density in its particles, whereas the application of TFUT results in particles of varying and irregular sizes.

The process of germination yields more distinctly defined uneven particles, while the combination of TFUT + GE treatment results in poorly dispersed particles of larger size ranges. This phenomenon can be ascribed to shear forces, microjets, and localized heating produced by sonication, which enhanced the sprouting effects on barley starch [67]. The soaking treatment yields particles that are larger and exhibit a non-uniformity not observed in alternative treatments. The findings indicateed that the interaction between ultrasound and germination, especially when combined, brings about noteworthy structural alterations that could influence material characteristics, including granule size distribution and the architecture of voids, with possible ramifications for processing and functional applications [68]. Our findings correspond with previous studies where authors observed a comparable pattern in grooves on granular surfaces resulting from varying grain germination [69]. Furthermore, these grooves could develop from partial hydrolytic breakdown during germination, as previously observed [70].The morphology of ZQ2000 deteriorated and became coarse during germination and soaking; however, the combination of ultrasound and germination had a more pronounced impact, with the order of efficacy being TFUT + GE > TFUT > GE > TS > UB.

### 3.10 Molecular docking

**Fig. 9:**
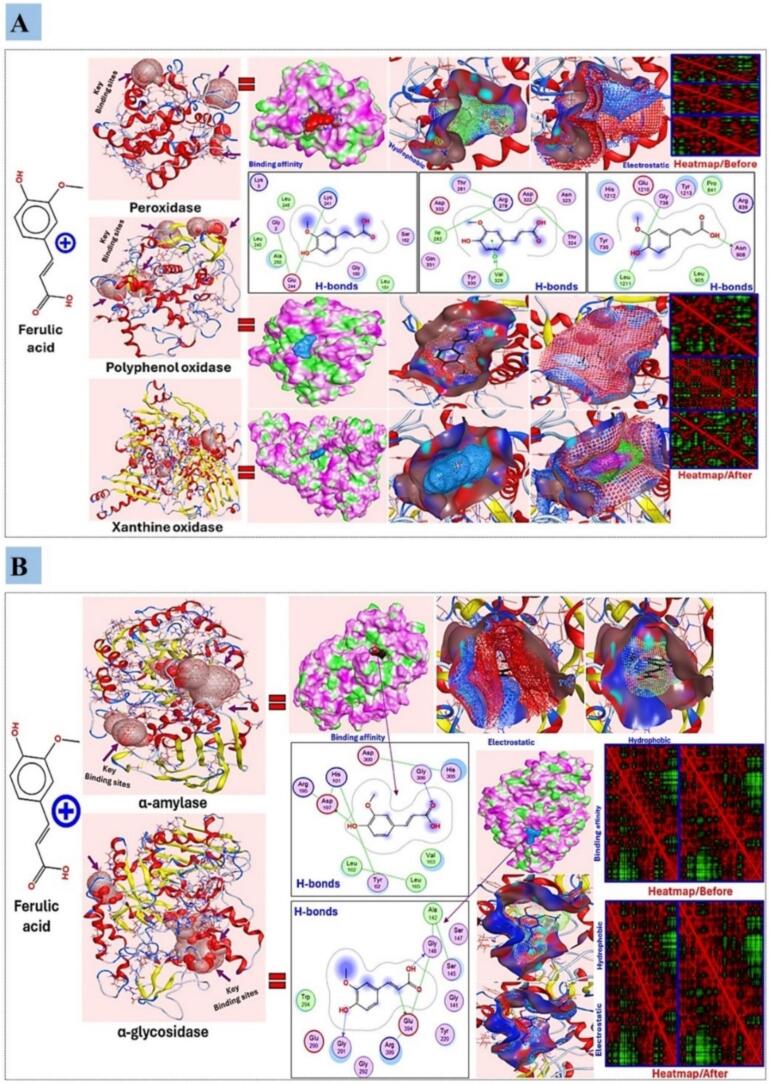
Interaction scheme of ferulic acid with three key oxidative enzymes (peroxidase (POD), polyphenol oxidase (PPO), and xanthine oxidase) (Fig. 9A) and carbohydrate hydrolyzing enzymes (α-amylase and α-glycosidases) (Fig. 9b) structures with representing their binding affinity, overall conjugates, hydrophobicity alterations, electrostatic potentials, H-bonding, and heat-map analysis. The end conjugates showed a binding score and energy affinity of −1.02 and −1.83 kcal/mol, separately.

Ferulic acid, a phenolic compound abundantly found in barley, has been studied for its antioxidant, anti-inflammatory, and antimicrobial properties. It has been suggested as a potential therapeutic agent due to its ROS scavenging and antioxidative enzyme regulatory effects [71]. The molecular docking analysis revealed similar interactions between ferulic acid and three key oxidative enzymes: peroxidase (POD), polyphenol oxidase (PPO), and xanthine oxidase (Fig. 9A). The interactions were influenced by hydrogen bonds and hydrophobic interactions, with ferulic acid preferring deep binding to catalytic residues in all three enzymes. Furthermore, the molecular interaction demonstrated the prevention of multiple H-bonds with vital amino acids. Ferulic acid’s hydroxyl and carboxyl groups formed H-bonds with Lys241, Leu151, Glu244, and Gly150, stabilizing the ligand-enzyme complex in peroxidase. Ferulic acid developed H-bonds with Thr281, Arg278, Asp322, Asn323, Val329, and Gln331 in polyphenol oxidase, which may affect enzyme function. Xanthine oxidase formed H-bonds to His1212, Glu1210, Pro841, Leu1211, Arg839, Tyr1213, and Asn908. These hydrogen bonding interactions were expected to assist ferulic acid in blocking polyphenol oxidase and xanthine oxidases, which promoted oxidative stress and ROS formation. Studying hydrophobic and electrostatic interaction surfaces confirmed their role in stabilizing ferulic acid-enzyme complexes. Ferulic acid was mostly found at the docking site’s hydrophobic pockets in both enzyme structures, according to electrostatic surface maps (Fig. 9A). Ferulic acid interacts with these areas, resulting in significant binding energy and affinity, according to this study’s docking poses. The non-polar interactions between Leu905 and Tyr735 (xanthine oxidase) suggest that hydrophobic residues may regulate enzyme activity. Heatmaps show that ferulic acid binding generated substantial conformational changes in the active regions of all three enzymes (Fig. 9A). These heatmaps show movement and interaction strength variations with ferulic acid. Ferulic acid binding to peroxidase generated large-scale structural changes, which may have affected catalytic activity. Both polyphenol oxidase and xanthine oxidase have changed active site designs, which may affect their ROS generation and ferulic acid’s antioxidant capabilities.

Moreover, this study found that ferulic acid has potent inhibitory activities against major carbohydrate hydrolyzing enzymes like α-amylase and α-glycosidases, which regulate postprandial blood glucose levels. This inhibition prevents postprandial hyperglycemia and helps alleviate diabetes mellitus. Molecular docking was used to explain the inhibition activities and elucidate the inhibitory mechanisms. Ferulic acid showed good binding ability with α-amylase and α-glycosidase based on the result of molecular docking (Fig. 9B). Ferulic acid binds α-amylase at crucial sites, including Asp197, His101, and Gly306 for intramolecular interactions. Ferulic acid binds to key subdomains of α-glycosidase, such as Glu290, Tyr220, Ala142, Ser147, Trp294, Arg399, and Gly146, forming stable connections with nitrogen atoms ((Fig. 9B). These interactions show that ferulic acid strongly inhibits both enzymes by blocking their active sites and distant complexes and restricting substrate accessibility.

Hydrogen bonding also stabilizes enzyme-ligand complexes. While α-amylase maintains several hydrogen bonds with Asp197, Tyr62, and residues like His305, complexes including Leu162 and Val163 provide further hydrophobic stability. Bonding networks to Ser145 and Glu394 in the α-glycosidase complex can be established, complementing the mechanism for stabilizing enzyme-ligand contact. Ferulic acid inhibits catalysis by locking the substrate in a stable bound phase through its vast hydrogen bond network in both complexes. The docking results demonstrated that ferulic acid bound to both enzyme structures via hydrophobic and electrostatic interactions, stabilizing their complexes. Pure ferulic acid was docked into the α-amylase complex, forming a hydrophobic pocket with residues Leu162, His101, and Val163. For α-glycosidase, ferulic acid binds to hydrophobic residues like Gly141, Ala142, and Ser147.

Heatmaps indicate that binding ferulic acid causes structural rearrangements in α-amylase and α-glycosidase ((Fig. 9B). Active site areas like ferulic acid binding show modifications that diminish flexibility and increase structural stiffness. Proper enzyme-substrate alignment precludes catalytic turnover; hence, this conformation constraint may explain the observed inhibition. Ferulic acid exhibits substantial binding affinity for α-amylase and α-glycosidase, forming a stable complex through hydrogen bond, hydrophobic interactions, and electrostatic force, as shown by molecular docking research. These results shed some light on the underlying antidiabetic mechanism of ferulic acid, and advocate its potential to be a natural carbohydrate-hydrolyzing enzyme inhibitor.

## 4 Conclusion

This work represents the first report on the synergistic effects of distinct treatments, namely UB, TFUT, GE, TFUT + GE, and TS, on the physicochemical, structural, and techno-functional characteristics of two barley varieties, ZQ2000 and XMLY22. The combined treatment of ultrasound and germination has proven to be highly effective in enhancing the germination rate and bioactive compound content in two barley varieties, ZQ2000 and XMLY22. This approach significantly increased TPC, TFC, and antioxidant activities, as demonstrated by improved DPPH, ABTS, and FRAP scavenging capacities. Additionally, the combined treatment boosted the enzymatic activities of POD and PPO, as well as elevated the levels of key antioxidant enzymes such as CAT and SOD. The treatment also resulted in higher γ-Aminobutyric Acid (GABA) content and showed potential antidiabetic properties through increased α-amylase and α-glucosidase inhibitory activities. Furthermore, gene expression analysis revealed upregulation of critical genes in the phenylpropanoid pathway, contributing to the enhanced accumulation of polyphenolic compounds. The study concludes that the combined TFUT and GE treatment offers a promising strategy for improving barley’s nutritional and functional properties, with potential applications in the development of health-promoting food products.

## CRediT authorship contribution statement

**Tabussam Tufail:** Writing – original draft, Investigation, Formal analysis, Data curation, Conceptualization. **Huma Bader Ul Ain:** Methodology. **Jawad Ashraf:** Writing – review & editing, Software, Methodology. **Muhammad Safiullah Virk:** Writing – review & editing, Visualization, Formal analysis. **Zahoor Ahmed:** Writing – review & editing, Software. **Mokhtar Dabbour:** . **Tawfiq Alsulami:** . **Suleiman Althawab:** Writing – review & editing. **Bin Xu:** Writing – review & editing, Visualization, Supervision, Resources, Project administration, Funding acquisition.

## Declaration of competing interest

The authors declare that they have no known competing financial interests or personal relationships that could have appeared to influence the work reported in this paper.

## References

[1] Obadi M., Sun J., Xu B. (2021). *Highland barley: Chemical composition, bioactive compounds, health effects, and applications*. Food Res. Int..

[2] Guo T. (2020). *Understanding the nutrient composition and nutritional functions of highland barley (Qingke): A review*. Trends Food Sci. Technol..

[3] Geng L. (2022). *Barley:a potential cereal for producing healthy and functional foods*. Food Qual. Saf..

[4] Elakhdar A. (2022). *Barley with improved drought tolerance: Challenges and perspectives*. Environ. Exp. Bot..

[5] Qiao Z. (2024). *Low frequency ultrasound enhanced the antioxidant activity and isoflavones accumulation of soybean sprouts by inducing oxidant stress*. Food Biosci..

[6] Jayakodi M. (2020). *The barley pan-genome reveals the hidden legacy of mutation breeding*. Nature.

[7] Sita K., Kumar V. (2020). *Role of Gamma Amino Butyric Acid (GABA) against abiotic stress tolerance in legumes: a review*. Plant Physiology Reports.

[8] Avdeeva L., Sh A.U., Ultrasound treatment: effect on germinating barley. (2023). Becтник Южнo-Уpaльcкoгo гocyдapcтвeннoгo yнивepcитeтa. Cepия: Пищeвыe и Биoтexнoлoгии.

[9] Didmanidze O. (2021). *IOP Conference Series: Earth and Environmental Science*.

[10] Silventoinen P., Sozer N. (2020). *Impact of ultrasound treatment and pH-shifting on physicochemical properties of protein-enriched barley fraction and barley protein isolate*. Foods.

[11] Yen N.T.H., Hoa P.N., Hung P.V. (2022). *Optimal soaking conditions and addition of exogenous substances improve accumulation of γ‐aminobutyric acid (GABA) in germinated mung bean (Vigna radiata)*. Int. J. Food Sci. Technol..

[12] Ahmed Z. (2024). *Impact of multi-frequency ultrasound processing with different treatment times on the structural quality of frozen wheat dough*. Ultrason. Sonochem..

[13] Liu Y. (2024). *Effect of GABA combined with ultrasound stress germination treatment on phenolic content and antioxidant activity of highland barley*. J. Sci. Food Agric..

[14] Liu S. (2022). *New perspectives on physiological, biochemical and bioactive components during germination of edible seeds: A review*. Trends Food Sci. Technol..

[15] Qin Q. (2024). *Enhancing malting performance of harder barley varieties through ultrasound treatment*. Ultrason. Sonochem..

[16] Grgić T. (2023). *Ultrasound-assisted modification of enzymatic and antioxidant activities, functional and rheological properties of oat and barley bran*. Food Bioproc. Tech..

[17] Memiş N. (2022). *Effect of ultrasonic treatment on seed germination and seedling emergence in seven vegetable species*. Anadolu Tarım Bilimleri Dergisi.

[18] Mariam E.-S., Ahmed M.A., Osman M.A. (2020). *Study of germination, soaking and cooking effects on the nutritional quality of goat pea (Securigera securidaca L.)*. Journal of King Saud University-Science.

[19] Li M.-J. (2022). *Effect of pearling on composition, microstructure, water migration and cooking quality of highland barley (Hordeum vulgare var. Coeleste Linnaeus)*. Food Chem..

[20] Tufail T. (2024). *Contemporary Views of the Extraction, Health Benefits, and Industrial Integration of Rice Bran Oil: A Prominent Ingredient for Holistic Human Health*. Foods.

[21] Gong M. (2020). *Effects of soaking on physicochemical properties of four kinds of rice used in Huangjiu brewing*. J. Cereal Sci..

[22] Bai Y.-P. (2021). *Effect of thermal treatment on the physicochemical, ultrastructural and nutritional characteristics of whole grain highland barley*. Food Chem..

[23] Shi P. (2019). *Total phenolic, flavonoid content, and antioxidant activity of bulbs, leaves, and flowers made from Eleutherine bulbosa (Mill.) Urb*. Food Sci. Nutr..

[24] Shraim A.M. (2021). *Determination of total flavonoid content by aluminum chloride assay: A critical evaluation*. Lwt.

[25] Dou J. (2022). *Application of exogenous melatonin improves tomato fruit quality by promoting the accumulation of primary and secondary metabolites*. Foods.

[26] Ilyasov I.R. (2020). *ABTS/PP decolorization assay of antioxidant capacity reaction pathways*. Int. J. Mol. Sci..

[27] Amoghin M.L. (2024). *Automatic non-destructive estimation of polyphenol oxidase and peroxidase enzyme activity levels in three bell pepper varieties by Vis/NIR spectroscopy imaging data based on machine learning methods*. Chemom. Intel. Lab. Syst..

[28] Reza S. (2006). *Antioxidant response of two salt-stressed barley varieties in the presence or absence of exogenous proline*. Gen. Appl. Plant Physiol.

[29] Cao K. (2022). *Effects of soaking and germination on deoxynivalenol content, nutrition and functional quality of Fusarium naturally contaminated wheat*. LWT.

[30] Ma Y. (2018). *GABA enhances physio-biochemical metabolism and antioxidant capacity of germinated hulless barley under NaCl stress*. J. Plant Physiol..

[31] Ge X. (2021). *The phenolic compounds profile, quantitative analysis and antioxidant activity of four naked barley grains with different color*. Food Chem..

[32] Sun L., Wang Y., Miao M. (2020). *Inhibition of α-amylase by polyphenolic compounds: Substrate digestion, binding interactions and nutritional intervention*. Trends Food Sci. Technol..

[33] Zhang G., Bown A.W. (1997). *The rapid determination of γ-aminobutyric acid*. Phytochemistry.

[34] Dutta S.D. (2021). *Effects of GABA/β-glucan supplements on melatonin and serotonin content extracted from natural resources*. PLoS One.

[35] Edalatian Dovom M.R. (2023). *Screening of lactic acid bacteria strains isolated from Iranian traditional dairy products for GABA production and optimization by response surface methodology*. Sci. Rep..

[36] Zhao F. (2021). *An optimized protocol for stepwise optimization of real-time RT-PCR analysis*. Hortic. Res..

[37] Rehman A. (2024). *Co-encapsulation of borage seed oil and peppermint oil blends within ultrasound-assisted soy protein isolate/purity gum ultra complex nanoparticles: Fabrication, structural interaction mechanisms, and in vitro digestion studies*. Food Chem..

[38] Khalifa I. (2023). *Covalently phenolated-β-lactoglobulin-pullulan as a green halochromic biosensor efficiency monitored Barramundi fish's spoilage*. Int. J. Biol. Macromol..

[39] Rehman A. (2025). *Localized enzymolysis and dual-frequency ultrasound modification of underutilized fava bean protein: Techno-functional, structural, in vitro digestibility, molecular docking, and interrelationship characteristics*. Food Hydrocoll..

[40] Zhao C.-C., Kim P.-H., Eun J.-B. (2020). *Influence of high-intensity ultrasound application on the physicochemical properties, isoflavone composition, and antioxidant activity of tofu whey*. Lwt.

[41] Wang J. (2019). *Influence of high-intensity ultrasound on bioactive compounds of strawberry juice: Profiles of ascorbic acid, phenolics, antioxidant activity and microstructure*. Food Control.

[42] Wang L. (2023). *Effects of pretreatment with a combination of ultrasound and γ-aminobutyric acid on polyphenol metabolites and metabolic pathways in mung bean sprouts*. Front. Nutr..

[43] Chiu K.-Y. (2021). *Changes in microstructure, germination, sprout growth, phytochemical and microbial quality of ultrasonication treated adzuki bean seeds*. Agronomy.

[44] Aadil R.M. (2013). *Effects of ultrasound treatments on quality of grapefruit juice*. Food Chem..

[45] Mustapha A.T. (2020). *Efficacy of dual-frequency ultrasound and sanitizers washing treatments on quality retention of cherry tomato*. Innov. Food Sci. Emerg. Technol..

[46] Sarker U., Islam M.T., Oba S. (2018). *Salinity stress accelerates nutrients, dietary fiber, minerals, phytochemicals and antioxidant activity in Amaranthus tricolor leaves*. PLoS One.

[47] Kowalczewski P.Ł. (2020). *Influence of Abiotic Stress Factors on the Antioxidant Properties and Polyphenols Profile Composition of Green Barley (hordeum Vulgare l.).* International Journal of Molecular Sciences.

[48] Punia S., Sandhu K.S. (2015). *FUNCTIONAL AND ANTIOXIDANT PROPERTIES OF DIFFERENT MILLING FRACTIONS OF INDIAN BARLEY CULTIVARS.* Carpathian. J. Food Sci. Technol..

[49] Lurie S., Watkins C.B. (2012). *Superficial scald, its etiology and control*. Postharvest Biol. Technol..

[50] Tang D. (2014). *Metabolomic analysis of the polyphenols in germinating mung beans (Vigna radiata) seeds and sprouts*. J. Sci. Food Agric..

[51] Noreen S. (2023). *Secoisolariciresinol diglucoside and anethole ameliorate lipid abnormalities, oxidative injury, hypercholesterolemia, heart, and liver conditions*. Food Sci. Nutr..

[52] Iqbal A. (2019). *Activation and inactivation mechanisms of polyphenol oxidase during thermal and non-thermal methods of food processing*. Food Bioprod. Process..

[53] Huang G. (2017). *Effects of ultrasound on microbial growth and enzyme activity*. Ultrason. Sonochem..

[54] Guo Y. (2021). *Ultrasonication and thermosonication blanching treatments of carrot at varying frequencies: Effects on peroxidase inactivation mechanisms and quality characterization evaluation*. Food Chem..

[55] Jacobo-Velázquez D.A. (2011). *Plants as biofactories: Physiological role of reactive oxygen species on the accumulation of phenolic antioxidants in carrot tissue under wounding and hyperoxia stress*. J. Agric. Food Chem..

[56] Liao J. (2017). *γ-Aminobutyric acid (GABA) accumulation in tea (Camellia sinensis L.) through the GABA shunt and polyamine degradation pathways under anoxia*. J. Agric. Food Chem..

[57] Podlešáková K. (2019). *Phytohormones and polyamines regulate plant stress responses by altering GABA pathway*. N. Biotechnol..

[58] Yang, R., et al., *Ca2+ and aminoguanidine on γ-aminobutyric acid accumulation in germinating soybean under hypoxia–NaCl stress.* journal of food and drug analysis, 2015. **23**(2): p. 287-293. 10.1016/j.jfda.2014.07.004 PMC9351771 28911384

[59] Liu M.-Q. (2024). *Regulation and mechanism of enzyme metabolism in germinated hemp seeds by ultrasound combined with exogenous calcium chloride treatment*. Int. J. Biol. Macromol..

[60] Ghani U. (2015). *Re-exploring promising α-glucosidase inhibitors for potential development into oral anti-diabetic drugs: Finding needle in the haystack*. Eur. J. Med. Chem..

[61] Mehmood A. (2019). *Management of hyperuricemia through dietary polyphenols as a natural medicament: A comprehensive review*. Crit. Rev. Food Sci. Nutr..

[62] Karim A. (2024). *Fabrication and Characterization of Sonicated Peach Gum-Sodium Caseinate Nanocomplexes: Physicochemical, Spectroscopic, Morphological, and Correlation Analyses*. Food Bioproc. Tech..

[63] Rehman A. (2025). *Co-encapsulation of borage seed oil and peppermint oil blends within ultrasound-assisted soy protein isolate/purity gum ultra complex nanoparticles: Fabrication, structural interaction mechanisms, and in vitro digestion studies*. Food Chem..

[64] Lou X. (2022). *Effect of ultrasound treatment on the physicochemical and structural properties of long-chain inulin*. Lwt.

[65] Manzoor M.F. (2019). *Combined impact of pulsed electric field and ultrasound on bioactive compounds and FT-IR analysis of almond extract*. J. Food Sci. Technol..

[66] Gani A. (2022). *Ultrasonication as an innovative approach to tailor the apple seed proteins into nanosize: Effect on protein structural and functional properties*. Ultrason. Sonochem..

[67] Bonto A.P. (2021). *Impact of ultrasonic treatment on rice starch and grain functional properties: A review*. Ultrason. Sonochem..

[68] Monroy Y., Rivero S., García M.A. (2018). *Microstructural and techno-functional properties of cassava starch modified by ultrasound*. Ultrason. Sonochem..

[69] Li C. (2017). *Effect of germination on the structures and physicochemical properties of starches from brown rice, oat, sorghum, and millet*. Int. J. Biol. Macromol..

[70] Claver I.P. (2010). *Impact of the soak and the malt on the physicochemical properties of the sorghum starches*. Int. J. Mol. Sci..

[71] Suriano S. (2018). *Phenolic acids profile, nutritional and phytochemical compounds, antioxidant properties in colored barley grown in southern Italy*. Food Res. Int..

